# On the Starched Bandage in Fractures

**Published:** 1842-12-17

**Authors:** 


### ANALECTA.

*On the starched Bandage in Fractures.*—M. Seutin, the eminent surgeon of Brussels, accompanied by Doctor Simonart, has just paid this city a visit. As is well known, he is the inventor of the use of the starch bandage in fractures. He availed himself of an opportunity afforded him by Mr. Cusack in Steevens' Hospital to put his apparatus on the leg of a little boy who had fractured both bones a fortnight before. Sir Philip Crampton, Mr. Cusack, and a number of Surgeons and pupils were present; M. Seutin stated how anxious he was himself to exhibit the application of the apparatus, because he had found that one great reason of its not having been more extensively used, nay, in some instances not even tried, was owing to its having been generally misunderstood. One great objection had been, that the apparatus, when once put on, remained a hard case round the limb, allowing no room for the necessary degree of tumefaction, and consequently endangering the safety of the member by inducing gangrene; that as the parts were hid from view, no timely warning was afforded of such accidents. As the starch bandage *was* used, there was much truth in this objection; as M. Seutin *now* uses it, the occurrence of such dangers is obviated. He first applied a calico roller, moderately firm, round the leg; no starch was put on the inside of this bandage, as it would stick in the hairs, and prove unpleasant to the skin when hardened. After it was applied, some starch was smeared along its surface; wherever pressure was wished to be avoided, pledgets of soft lint were put; a soft pasteboard splint, a little starched on the inside, was then placed on each side of the leg, and then one behind, the part about the heel and the hollow of the tendo achillis being well stuffed with lint; a pasteboard splint was also then put in front. These were secured by a bandage smeared with starch, the end of the bandage being turned down and stuck in front, so as to be easily found. More starched bandage was applied, till the whole was a firm and smooth case. This should be left for twenty-four hours; when it has become quite dry, it is then slit down along the whole front of the outside in the space between the tibia and fibula, down to the end of the foot; when the sides of this opening are held aside, the state of the limb can be examined. If it is found to press too much on any part, a little lint can be inserted, so as to raise the apparatus from the place pressed on: should it be desirable, any part of it covering a wound, &c. can be cut away, to allow the proper dressings to be applied, and the discharge to get out. Modified as it now is, we cannot but think, that M. Seutin will have the gratification of seeing his method generally used. We insert the accounts given by his pupils, MM. Simonart and Porcelet, of the circumstances to be observed in the application of the starch bandage.

"In the construction of the starch bandage,* use is made of the bandages of Scultetus, long or short, made of half-used linen, neither too coarse nor too fine, replaced if necessary in the central layers, by rather long compresses, extended the length of the splints, or even by the immediate application of pasteboard on the limb. Long bandages are preferred, wherever it is requisite to establish a regular compression, and that the lifting of the injured part cannot entail inconvenience to the patient, sharp pain, derangement in the coaptation, &c. Short bandages are reserved for contrary cases; they are disposed generally in three planes; it is between the layer in contact with the skin and the middle layer, that the pasteboard splints are generally placed; short bandages are especially employed in lesions of the pelvic extremity. The length or breadth of the bandages is proportioned to the part which ought to be covered by them. From the bandage which is applied immediately to the skin, more care is used than in the common bandage, to remove coarse edges, folds, irregular plaits; folds should be repeated as seldom as possible; these are kept as far as can be from the bony eminences, from excrescences, &c., which are first defended by some soft material, a layer of wadding, lint, &c., placed over or around these eminences, that besides are not entirely covered by all the bandages. These last precautions have for object to diminish the degree of pressure that the bandage could exercise more on them than on the soft parts, whose level these eminences exceed. Great care is taken that the bandages, and the containing apparatus generally, do not establish strangulation in some point of the length of the limb.

"All these minute cares, just described, are less to be enforced in applying the superficial layers of the bandage; in the case of penury, linen may be replaced by remnants of handkerchiefs, aprons, towels, &c.; often in fractures of the leg, when walking is authorized, we surround above the level of the ankles, the dried bandage with bandages composed of old handkerchiefs, intended to maintain the efficiency of the apparatus.

"A very useful precaution is, that when the bandage is exhausted, the end should be folded or turned down on itself to a certain extent, and stuck so in a conspicuous part of the bandage, so that if it should be necessary to remove the apparatus, no difficulty should be experienced in finding the end of the bandage.

* The following is the interesting account of what first led M. Seutin to the use of the starch bandage; it is not the first time mankind has been indebted to animals for valuable therapeutic agents: "A lady of this city was very fond of a goat, which one day broke its paw, while I was in the house; she besought me to relieve the poor animal; I willingly consented, but I was very much embarrassed when the question of contriving some means to keep the part fixed presented itself, for nothing offered fit to keep the fragments together. While I was thinking what to do, I observed an old piece of rope belonging to a well, and some starch which was for stiffening linen. I availed myself of these two means which chance presented to me, and I constructed on the spot with them a bandage, which I was astonished to find some days after perfectly adapted to the case. This circumstance opened my eyes to the use that might be drawn from starch in the therapeutics of the affections with which I had been occupied for some time, and it is thus that my experiments began on the bandage, which has since rendered me much service."

"Also, always take care to leave uncovered the ends of the fingers or toes, whose variations of colour and of temperature furnish you with a sufficiently just measure of the analogous changes of the other parts of the limb covered by the bandage. Does a suppurating wound, or one that we cannot prevent passing into suppuration, exist on the injured limb, either the turns of the bandage are so arranged, that the solution of continuity of the soft parts is not completely covered by them; the edges of it are either cut out or turned back and stuck so with the starch; the bandage is fully rolled round the wound of which it takes the impression, marked by the blood or pus, it is raised by means of bent scissors, or of a cutting nippers (*emporte piece.**) One or several openings are cut in the central part, and as much as possible in the longitudinal direction; these openings should be large enough to allow a passage for the secretions, but not so large as to permit a protrusion and consecutive strangulation of the fleshy granulations.

"The compression made by the starch bandage ought never to reach, and especially in fractures, that degree of violent constriction that practitioners, as little familiarized with M. Seutin's method as with the general principles of compression, have believed to be necessary for the resolution or prevention of inflammation. A like error can only expose to a rapid disorganization, to accidents too often set down to the effects of the starch bandage, and which, in our opinion, ought rather to be attributed to the surgeon who does not judiciously regulate the application of a means so essentially useful in other hands. *Compression, as understood by M. Seutin, ought to stop at a gentle, methodical pressure, sufficient to moderate the afflux of blood, but not to stop it, a pressure which in many circumstances, at the instant of its application, is only retentive, and which never acts on the soft parts, so as to be able to induce mortification in their tissues*, and even in fractures we frequently see the clinical professor apply the first layers of bandages, in a manner simply retentive; those which support the splints only make a slight degree of pressure, particularly near the seat of the fracture. In complicated fractures, we have often heard him repeat to his pupils, the express recommendation to employ only a very moderate compression, particularly the first few days, and even in severe cases to dispense with it completely, adding, that tissues lacerated, violently contused, or under the influence of of a violent shock, would more than ever tend to disorganization, if in the period of reaction, the least exterior cause should join itself to their already dangerous tendency. In old men, wounded cathetic subjects, the precautions should be even yet more minute.

"Practice alone will tell to what degree compression must be carried or restricted; theory can only guess. It should always be made to act from the extremities to the centre, as evenly as possible, care being taken to avoid its immediate action on bony or tendinous prominences, or excrescences, &c. The filling up is formed by means of compresses, folded on themselves, or enclosing old linen, tow, charpie, wadding, cotton, &c.; they can also be formed of soft pieces of pasteboard put one over the other, and fitted to the hollow that they are destined to fill. The starch keeps together the edges, or the opposed surface of the fillings up.

* M. Seutin has invented a scissors of a particular construction, for the purpose of setting up the apparatus when dried, or cutting out such parts of it as may be necessary. Mr. Milliken, the cutler, of Grafton street, has the pattern of it, and is making one by M. Seutin's direction.

"The starch paste ought to be, if possible, freshly prepared, and without lumps. Starch lightly boiled (in an iron saucepan without the lid) has appeared to us to present all the advantages of dextrine, applied in the manner of M. Velpeau, without partaking the disadvantages that this substance presents. Like it, it dissolves in cold water, readily imbibes the bandages plunged into its solution; it is cheaper, and not so scarce, and does not make the bandages stick too closely together, so that when it is necessary to detach them they tear, if water has not penetrated all the layers of the bandage.

" Notwithstanding these qualities of boiled starch, M. Seutin nearly constantly employs the amylaceous fecula not boiled, which offers the great advantage of being met with every where, and not to demand much preliminary preparation. It is first diluted with cold water, then with boiling water, as is done by washerwomen. He prefers this simple solution, to that united to plaster, lime, alum, acetate of lead, Flanders' glue, &c. He also gives it the preference to the white of egg, or the composition of M. Larry, to flour, fresh plaster, turpentine, isinglass, resin, pitch, the gums, the gum-resins, the different mixtures in which resin enters, either as base or as adjuvant. Comparative trials of these different substances have led to this choice. The starch paste can be spread by means of a brush, or of the hand. The brush is indicated, when every motion should be avoided, or when both hands of the surgeon should remain free and not sticky, while putting on the apparatus. If it is desirable in stiffening the bandage to use the hand, the hollow of the palm, covered with starch, is carried flat, successively from the bottom to the top, and *vice versa;* then circularly, so that the edges of the bandage overlapping each other, may be first raised and starched, then flattened down again, and the inequalities effaced. A method of stiffening the bandage, as expeditious as commodious, and often employed by M. Seutin, consists in taking in the hollow of the hand a certain quantity of starch concreted by the cold; as the bandage is unrolled the surgeon makes it pass lightly over this substance, from which it takes at each turn a portion, which makes its turns stick together, and to the planes either over or under it. A light compression of the bandage effaces the irregularities which the starch might present in its dissemination.

" Whatever plan is followed, we should carefully avoid to starch the inside of the layer of bandage immediately applied to the skin, in order not to expose this last to a rough contact, a frequent source of smart itching. When the bandage is all applied, its surface is thinly starched. Care should be taken not to starch on the folds over joints, over bony prominences, or excrescences, nor on those edges of the bandage which may exercise any friction on the skin, and soon bring on excoriations, always disagreeable, often even very painful. The external layers of the apparatus should be freely starched.

" *Splints.*—Pasteboard is almost all that is necessary. It should offer sufficient resistance; a thickness of a line to a line and a half, but not to be too compact, so that water may readily penetrate it. If necessary, a portion of a hat box, book cover, &c., may be used instead. It is always better to tear it than to cut it, so that the edges, which only end by degrees, may adapt themselves more uniformly to the convex surface of the limbs, that the bandage may act gradually on all the surface of the splint; and to avoid troublesome impressions on the skin, which the compression of the bandage on the too regular edges of the splint, that is made by cutting, never fails to induce. The shape of the splints is regulated according to the case. When the splint is fit for application, it is lightly moistened, by making it pass quickly through tepid water, or plunging it some minutes in cold water; in this manner, although flexible, the splint yet preserves a certain degree of firmness. It is accurately applied to the convex surface of the parts, by bending it slightly and longitudinally on itself, in several parts of its surface. Its power of being softened makes it readily adapted to the irregularities of the most unfavorable surfaces to which it is applied. In most cases, especially in fractures, the external surface of the splints alone is spread with the starch, which is spread on it in a thin layer. If it is thought expedient to increase the firmness of the splints, one is stuck over the other, after having moistened them separately.

"A precaution essentially useful is, to prepare beforehand splints of pasteboard, broad and long, to soften them a little in water, to cover them freely with starch on both surfaces, then to leave them to dry slowly in the open air. Pieces of pasteboard so prepared are called *attelles de precaution*. It is very useful for the surgeon always to have a certain quantity of these splints ready. When wanted, it is sufficient to proportion the length and breadth of them to the dimensions of the injured part; to soften them only a little, and if they are only just moistened, they can reinforce the last bandage with a solid cuirass, which, covered with a starch bandage, maintains the fracture fixed, during the period of the dessication of the apparatus. Common splints of wood, tin, &c., may be used in the absence of these, particularly where there is a strong tendency to displacement on account of muscular spasm. The drying of the starch bandage is generally accomplished in thirty or forty hours after its application. This period may be shortened by artificial means, such as stone jars filled with hot water, or bags full of hot sand placed the length of the apparatus; exposure to the heat of the sun, of a stove, &c. Unless from urgent circumstances, it will be prudent to dispense with them in cases of complicated fracture. Unless the patient complains of pain, or much uneasiness in the injured limb; and the surgeon does not entertain reasonable fears on the state of the soft parts, it is not generally, in fractures, from the second to the fourth day that the section of the starch bandage is made. It is done with a pair of scissors invented by M. Seutin for the purpose. The section having been made, the state of the parts is to be attentively examined, and our conduct is regulated by the indications afforded by this inspection. If the apparatus fulfils the views proposed, it is made secure again by a starch bandage. Does it exercise too powerful a general pressure? relax it by contriving a separation between the edges, a suitable interval which is to be covered with a little plate of pasteboard, well softened and applied on the skin. The exterior surface of the bandage is then smeared with a coating of starch, the borders of the valves being properly held by the assistants, who raise at the same time the limb; the apparatus is then surrounded by a starched bandage, very little compressive. Folds and plaits that press the skin irregularly are to be removed; the pieces that exercise injurious local impressions are to be wet slightly with water; pieces of lint are inserted when necessary, and then the whole is surrounded by the starch bandage, care being taken to make a daily inspection to see that all is right.

"If the apparatus appears defective in any particular, do not hesitate to remove it (after having wet it with tepid water), to replace it by another, less objectionable.

"However it be, the section of the bandage has always appeared to us to be preferable, on account of the little shaking which it communicates to the injured limb, of the facility which it leaves for its inspection, and to fulfil all the indications which may offer themselves to the examiner. Thus the longitudinal division being finished, if we remark an evident tendency to suppuration, or if the purulent secretion has already established itself; and that during the application of the apparatus, we have not made in the bandages narrow openings in the situation of the wound, to permit a passage to the secretions furnished by them, a measure which ought always to be followed when suppuration is inevitable, if a like precaution has not been taken, either we make, by means of a pointed scissors, of a bistoury, penknife, &c., one or two holes in the bandage in the site of the wounds, or we make with M. Seutin's scissors, from the longitudinal section, two transverse divisions in the starched case, one above, the other below the level of the wound, divisions which can be repeated on the other side in the case where the suppurating surface is covered by it also.

"With respect to allowing the patients to walk about, in the surgical injuries of the thoracic extremity the wounded do not in general remain in bed, except in the case of some severe complication. In the pelvic extremity, it is only after the third day, in the majority of cases, that walking can be authorized, and that after the complete drying of the apparatus is assured, and that the section of the bandage has furnished satisfactory indications. In complicated fractures, and other violent lesions of the lower extremity, we wait for, before allowing it, the resolution of the first accidents.

"In the lesions of the pelvic extremity, walking ought always to be assisted by crutches, which are covered at their lower end with a piece of cloth to prevent their slipping; by a tarso-cervical suspensory, strong, and suitably tense, and, until the patient has acquired, in walking with crutches, the desirable habit and security, by a vigorous assistant who watches his steps, and supports him at the loins by raising the clothes there.

"*Regimen of fractured Patients.*—In simple fractures the regimen is not severe; the series of local antiphlogistics usually employed (emollient and discutient fomentations, leeches, poultices, &c.,) is excluded. In compound fractures, it is confined, in the generality of cases, to increase the degree of elevation generally given to the injured limb, the body being subjected to the horizontal position, the head excepted; to a rigorous diet; to large general bleedings, cooling drinks, intestinal derivatives, and where continual irrigations of cold water were indicated, we have seen on two occasions M. Seutin apply bladders filled with ice, the length of the fractured part, surrounded with a starched bandage. The two patients were cured."—***Dublin Journal of Medical Science, November***, 1842.

